# Genetic Diversity and Population Structure of Doum Palm (*Hyphaene compressa*) Using Genotyping by Sequencing

**DOI:** 10.3389/fgene.2022.762202

**Published:** 2022-02-04

**Authors:** Agnes Omire, Johnstone Neondo, Nancy L. M. Budambula, Laura Wangai, Stephen Ogada, Cecilia Mweu

**Affiliations:** ^1^ Department of Botany, School of Biological Sciences, Jomo Kenyatta University of Agriculture and Technology, Nairobi, Kenya; ^2^ Institute for Biotechnology Research (IBR), Jomo Kenyatta University of Agriculture and Technology, Nairobi, Kenya; ^3^ Department of Biological Sciences, School of Pure and Applied Sciences, University of Embu, Embu, Kenya; ^4^ Department of Biomedical Sciences, School of Health Sciences, Kirinyaga University, Kerugoya, Kenya

**Keywords:** genetic diversity, GBS, single nucleotide polymorphisms, population structure, *Hyphaene compressa*, doum palm

## Abstract

Doum palm (*Hyphaene compressa*) is a perennial economic plant primarily growing in Kenya’s Arid and Semi-Arid Lands (ASALs). It is heavily relied upon for food, animal feed, construction materials and medicine, making it an ideal plant for resource sustainability. However, the limited information on its genetic resources has hindered its breeding and conservation studies. This study used the genotyping by sequencing approach to identify Single Nucleotide Polymorphisms. These SNPs were further used to assess the genetic diversity and population structure of 96 *H. compressa* accessions from Coastal, Northern and Eastern ASAL regions of Kenya using two approaches; reference-based and *de novo*-based assemblies. STRUCTURE analysis grouped the sampled accessions into two genetic clusters (Cluster 1 and Cluster 2). Cluster 1 included accessions from the Northern region, whereas Cluster 2 included all accessions from Eastern and Coastal regions. Accessions from Kwale (Coastal) had mixed ancestry from both Cluster 1 and Cluster 2. These STRUCTURE findings were further supported by principal components analysis, discriminant analysis of principal components and phylogenetic analysis. Analysis of molecular variance indicated greater genetic variation within populations (92.7%) than among populations (7.3%). An overall F_ST_ of 0.074 was observed, signifying moderate genetic differentiation among populations. The results of this study will provide information useful in breeding, marker-assisted selection and conservation management of *H. compressa.*

## Introduction

*Hyphaene compressa* H. Wendl., also known as the East African doum palm, is a member of the Arecaceae family. It is integral in the agroforestry system of coastal and riverine parts of Africa (Amwatta, 2004; Uhl and Moore, 2019). The doum palm also grows in the arid and semi-arid lands (ASALs) of Kenya (Maundu and Tengnas, 2005). It is a valuable source of food, animal feed, medicine for headaches and worms, as well as non-wood products for construction and weaving. Thus, it is a substantial income-generating plant, particularly for women in ASALs who derive their livelihoods from the sale of woven leaf products (Amwatta, 2004; Maundu and Tengnas, 2005; Omire et al., 2020a). The ability to withstand waterlogging, drought and salinity makes the doum palm a reliable economic plant with ability to avert natural calamities including drought in such areas (Amwatta, 2004; Omire et al., 2020a).

In Kenyan ASALs, non-timber products are restricted to a few plant species such as *H. compressa*, subsequently threatening its existence. Thus, *H. compressa* is classified as a threatened and a national priority species in the ASALs of Kenya with a high potential for genetic erosion due to overexploitation by the rural communities (Kigomo, 2001). However, the International Union for Conservation of Nature (IUCN) considers it a species of least concern with an unknown population trend due to its wide geographical distribution throughout East Africa (Cosiaux et al., 2017). Whereas species might exist as large populations they could be regionally threatened. *Hyphaene. compressa* resources are known to be strained and overexploited in the Eastern part of Kenya (Omire et al., 2020a). Despite this knowledge on the status, there are no known interventions to reverse the current trend (Kigomo, 2001). This could exacerbate the risk of extinction of such species (Cosiaux et al., 2018).

Threats to *H. compressa* include human interference as well as biotic and abiotic stress (Omire et al., 2020a). Overgrazing by pastoralist communities, particularly along the riverine areas, is a significant threat to this palm since livestock graze and browse on *H. compressa* (Kigomo, 2001). The strain on *H. compressa* resources has been aggravated by the sedentarization of the nomadic pastoralists (Amwatta, 2004). Sedentarization leads to the assemblage of pastoralists around limited resources and ultimately to land degradation (Johnson, 1993). Another source of pressure on *H. compressa* is overharvesting and harvesting of immature sword leaves. These leaf pressures have been shown to cause dwarfing in a sister palm, *Hyphaene thebaica* (Kahn and Luxereau, 2008). Other selection pressures on *H. compressa* include logging, burning and wine tapping from the apical meristem. These pressures collectively lead to genetic drift through the loss of specific genotypes, which might eventually affect the *H. compressa* gene pool (Kigomo, 2001).

There is scanty information on the genetic diversity of doum palm which limits access to its important traits and thus hinders its improvement. Previous diversity studies on *H. compressa* focussed on accessing the variability of its morphological traits (Omire et al., 2020b). The study identified five morphotypes with accessions from the Kenyan Coastal area being the most heterogeneous. However, this cannot be used to adequately delineate the doum palm since morphological markers may also be affected by the environment, are limited in number, unstable, slow and some appear late in plant development making them difficult to score (Andersen and Lubberstedt, 2003; Mokhtar et al., 2016). Furthermore, using a single marker like morphology is not adequate to assess diversity (Khan et al., 2012). Overall, genetic markers are superior to morphological markers (Ganie et al., 2015) and may or may not agree with phenotypic expression of a genomic trait.

For non-model plants like doum palm with no reference genome, sequencing the whole genome to mine the SNPs would be ideal. There are other methods like Genotyping by Sequencing (GBS) that are able to acquire in depth data on parts of the genome and are as effective but less costly compared to whole genome sequencing (Wallace and Mitchell, 2017). GBS is a method that provides reduced libraries for Illumina next generation sequencing (NGS) by targeting the subsets with restriction enzymes followed by ligation of DNA barcoded adapters (Elshire et al., 2011). PCR amplification and high throughput NGS of the genomic subsets on a single lane of flow cells is then done (Elshire et al., 2011; He et al., 2014; Burghardt, et al., 2017). GBS is simple, rapid and highly reproducible (Davey et al., 2011; Burghardt et al., 2017). These features make GBS highly attractive for several genetic applications, including genetic diversity, phylogeny, genome-wide association studies, association maps, genomic selection, physical and linkage maps (Burghardt et al., 2017). GBS is an ideal tool for genetic diversity studies with the advantage of being able to identify SNPs, insertions, deletions and microsatellites (Elshire et al., 2011) even in non-model organisms with no prior genome information (Taranto et al., 2016). Diversity studies can be combined with phylogenetic studies to provide more information on the origin and domestication of the germplasm for conservation purposes (Burghardt et al., 2017). Earlier studies have alluded to the fact that the evolution of a population is guided by its local interactions in the environment (Klimova et al., 2018). Gene flow has a tendency to homogenize populations and reduce genetic variability (Brunet et al., 2012). However, there needs to be enough gene pool on which selection can take place for effective speciation.

Thus far, the genetic diversity of *H. compressa* remains unknown despite its economic and subsistence role in Africa’s ASALs. There were no *H. compressa* or other palms in the genus *Hyphaene* with assembled genomes at the time of this study. Due to the scanty genetic information coupled with the pressure already demonstrated on this palm, there is a need to decipher its genetics. This study assumes that the different accessions of *H. compressa* growing in Kenya are diverse. The present study aimed to identify genome-wide SNPs, assess the level of genetic diversity, determine the population structure and estimate gene flow between *H. compressa* accessions collected from four ASAL regions of Kenya using GBS approach. The data obtained from this study will be an important genomic resource that will be used to inform the conservation, management, breeding and propagation of *H. compressa*.

## Materials and Methods

### *H. compressa* Plant Materials

**FIGURE 1:**
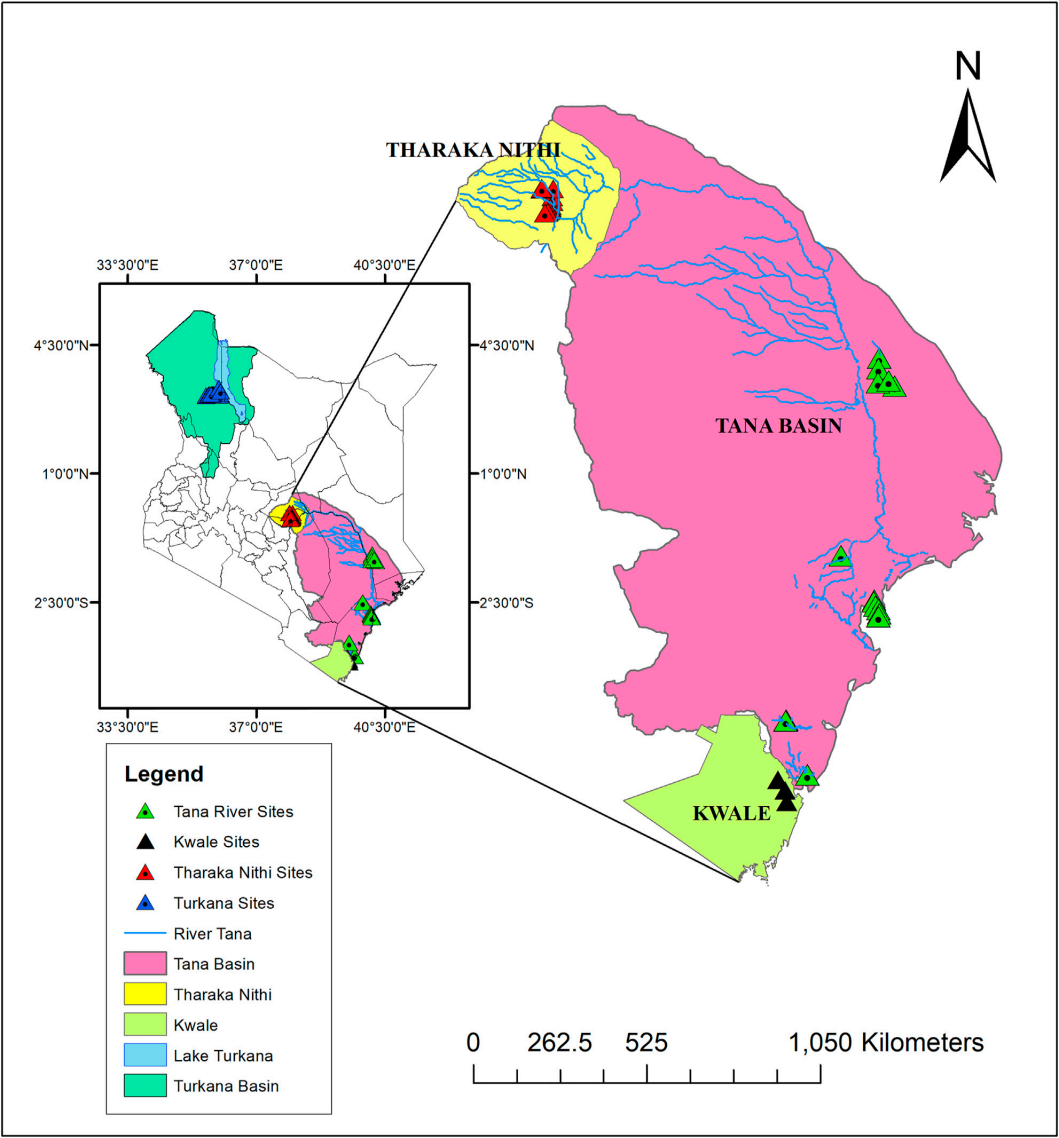
Map illustrating *H. compressa* sampling points along the Turkana and Tana basins. Map was created using ArcMap version 10.8.

A total of 96 *H. compressa* accessions collected between February and August of 2018 were used in this study. These accessions were collected from different ASALs of Kenya; Eastern (Tharaka Nithi County), Northern (Turkana County), Coastal (Kwale and Tana River counties) as shown in Figure 1 and Supplementary Table S1. Leaf samples of the selected plants were collected using sterile blades and placed in sterile tubes containing 10 g of silica gel for DNA extraction. Accessions within approximately the same age group and located as distantly as possible from each other were sampled.

### Preparation of Libraries and Sequencing

Genomic DNA was isolated from *H. compressa* leaves using DNeasy^®^ Plant Mini Kit (Qiagen, Germany). The purity and quantity of the DNA were determined using Qubit Fluorometer (Invitrogen) or microplate reader (DR-200B, Diatek), while a 1% agarose gel was used to confirm its integrity. Commercial GBS sequencing was done at Beijing Genomics Institute (BGI, China). A total of 96 samples; Tharaka (27), Turkana (21), Tana River (20) and Kwale (28), passed the sample quality check (QC) and proceeded to library preparation.

Library preparation was done following the method previously described by Elshire et al. (2011). Essentially, the DNA samples were barcoded and adapter pairs plated. The restriction enzyme ApeK1 (GCWGC as the recognition site) was used, followed by adapter ligation to the DNA fragments. This was followed by pooling and purification. PCR was then performed using primers with adapter binding sites. Sample clean-up of the PCR products, fragment size selection and sequencing on a Hiseq X10 platform as paired-end 100 bp (Illumina PE 100) was done. Adapter sequences, sequences with low-quality reads, and those lacking barcodes were discarded from the raw reads.

The data was processed using the *de_novo* and reference-based approaches. In the *de_novo* assembly, ipyrad version 0.9.74 (Eaton and Overcast, 2020) was used to assemble sequences without a reference genome using the following parameters; assembly method *de_novo*, datatype pairgbs, mindepth_statistical 6, mindepth_majrule 6, min_samples_locus 4 and other parameters set to default. In the reference-based approach, paired read ends were mapped to the *Phoenix dactylifera* (date palm) genome (Hazzouri et al., 2019). A confamilial (same family) reference genome was used for SNP calling (Galla et al., 2019) since *H. compressa* had no assembled genome at the time of this study. These two palms belong to the same subfamily Coryphoideae. *Phoenix dactylifera* genome was the only available genome in this subfamily. Alignment of the sequence reads against the date palm reference genome was done using the Burrows-Wheeler Aligner (BWA) using the parameters ‘mem–t4 –k32 –M’ (Li and Durbin, 2009). SNP filtering was done using VCFtools version 0.1.16 (Danecek et al., 2011) with the following parameters; biallelic SNPs, min meanDp 2, removing indels, Minor Allele Frequency (MAF) 0.05, minDP 2, max-missing 0.8.

### Data Analysis

The quality of the filtered VCF files were assessed using the R package tidyverse (Wickham et al., 2019). The read depth per site, heterozygosity, read depth per individual and read quality were determined.

### Population Structure Analysis and Genetic Diversity

Population structure was determined by STRUCTURE software version 2.3.4 using the admixture model (Pritchard et al., 2000). Populations of *K* (*K* = 1–10) were run with three replications using a burn-in of 100, 000 generations and 100, 000 Markov Chain Monte Carlo (MCMC) iterations. The software STRUCTURE HARVESTER web Version 0.6.94 (Earl and VonHoldt, 2012) available at http://taylor0.biology.ucla.edu/structureHarvester/ was used to determine the optimal K value using the *adhoc delta K* (Evanno et al., 2005). To plot the structure results, the POPHELPER version 2.3.1 package in R was used (Francis, 2017). Genotypes that had ≥0.80 membership proportion and those with less than this value were assigned to pure and admixture populations, respectively (Nkhoma et al., 2020).

Discriminant Analysis of Principal Components (DAPC) was also used to evaluate the population structure of *H. compressa* using the package adegenet version 2.1.3 (Jombart, 2008) in R. To visualize each sample’s assignment, a composite stacked bar plot illustrating the probability of population membership on the *Y*-axis was generated. Principal component analysis (PCA) was constructed using the R software package SNPrelate (Zheng et al., 2012) to determine the genetic relationships of *H. compressa* accessions.

Observed heterozygosity (*H*_*o*_), Expected heterozygosity (*H*_*e*_) fixation index (*F*_*ST*_), inbreeding coefficient (*F*_*IS*_), Analysis of Molecular Variance (AMOVA) and pairwise *F*_*ST*_ values of the population were determined using Arlequin version 3.5.2.2 (Excoffier and Lischer, 2010).

### Phylogenetic Analysis

To construct a splitstree, the filtered VCF file was converted to a nexus file using vcf2phylip.py script (Ortiz, 2019). The nexus file was then used to generate an unrooted splitstree using the neighbor net method in SplitsTree software, version 4.17.0 (Huson and Bryant, 2006). An UPGMA distance tree was also constructed using R software to represent the genetic clustering of *H. compressa* accessions.

### Migration Rates Between the Eastern and Coastal Populations Along the Tana Basin

To determine if the population structure observed along the Tana basin is influenced by seed dispersal along the river, gene flow was estimated using MIGRATE-n software version 3.6.11. A Bayesian inference strategy was used with constant mutation rates among all loci. Burn in was set at 5,000 iterations at each locus. Static heating at four different temperatures (1, 1.5, 3 and 6) was used to improve the MCMC searches. One gene flow model was designed with direct migration from Tharaka to Tana River and to Kwale. The drainage of the Tana basin was used to design this model. Turkana accessions were excluded from this model since structure analysis and PCA demonstrated little historical gene flow. To judge whether the runs converged on good answers, the histograms and the effective population sizes were checked.

## Results

Paired-end sequencing of 96 *H. compressa* accessions yielded an average of 2.4 million reads per sample. The *de_novo*-based assembly using ipyrad software resulted in 3,941 raw loci. After filtering, a total of 2,096 SNPs with a mean depth of 35.7 (minimum 10.47, maximum 217.45) were retained using the *de_novo* based assembly. On the other hand, reference-based assembly using date palm as a reference genome resulted in 3.4 million loci. After filtering, 23,146 biallelic SNPs with a mean depth of 3.5 (minimum 2, maximum 47.49) were obtained using the reference-based assembly.

The SNPs obtained from *de_novo* based assembly had higher depths than the reference-based assembly, as shown by the individual sequencing depth and the mean depth. The proportion of missing data per accession was low for both the *de_novo* based and reference-based assemblies, with a maximum of 0.04 and 0.4, respectively. These VCF quality statistics, including the mean depth, observed heterozygosity, depth per individual and missing data per individual, are presented for both the *de_novo* based assembly (Supplementary Figure S1) and reference-based assembly (Supplementary Figure S2).

In the *de_novo* based assembly there were 1,283 (61.2%) transition SNPs and 813 (38.8%) transversion SNPs with the following types: A↔G type (651, 31.1%), C↔T type (632, 30.2%), A↔C type (192, 9.2%), A↔T type (174, 8.3%), C↔G type (222, 10.6%), G↔T type (225, 10.7%). There were 16,598 (70.9%) transition SNPs and 6,818 (29.1%) transversion SNPs with the following types: A↔G type (8,332, 35.6%), C↔T type (8,266, 35.3%), A↔C type (1,684, 7.2%), A↔T type (1825, 7.8%), C↔G type (1,636, 7%), and G↔T type (1,673, 7.1%) in the reference based assembly. The A↔G and C↔T transition SNPs had the highest counts for both assemblies (Supplementary Table S2). The transition SNPs versus transversion SNPs (Ts/Tv) ratio was 1.6 in the *de_novo* based assembly and 2.4 in the reference based assembly.

### Population Structure and Genetic Diversity

**FIGURE 2:**
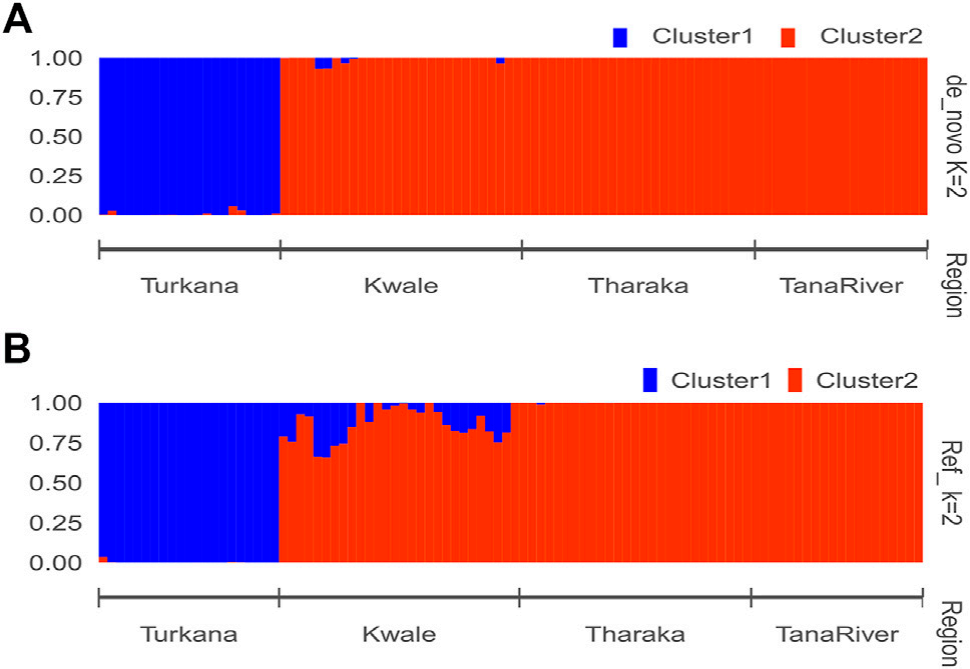
STRUCTURE bar plot of admixture model population assignment of 96 *Hyphaene compressa* accessions from Kenya based on 2,096 SNPs for the *de_novo* assembly **(A)** and 23,416 SNPs based on reference assembly **(B)**. The accessions are divided into two clusters. A combination of different colors represents admixed populations.

**FIGURE 3:**
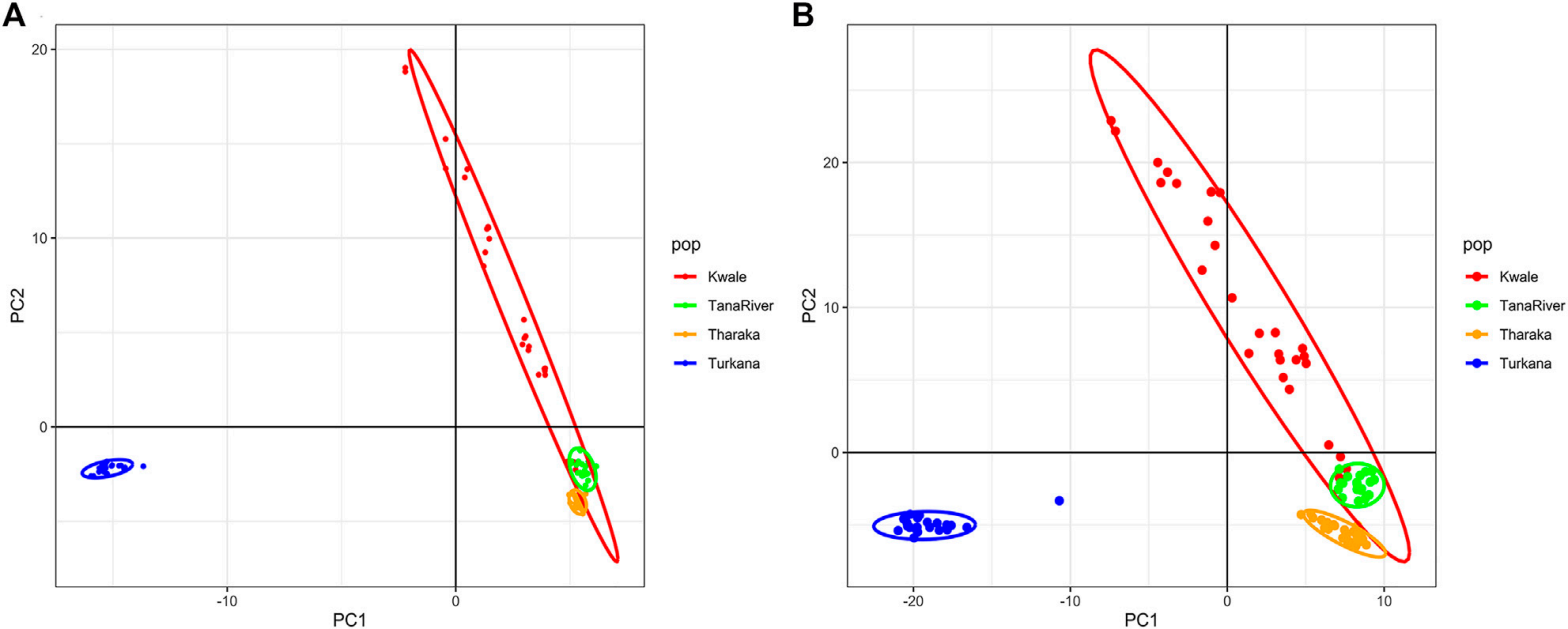
Principal Component Analysis (PCA) of *Hyphaene compressa* Kenyan accessions using 2096 SNPs obtained from the *de novo* assembly **(A)** and 23,416 SNPs obtained from the date palm reference based assembly **(B)**.

The optimal delta *K* was detected at *K* = 2 for both the *de_novo*-based assembly (Supplementary Figure S3A) and reference-based assembly (Supplementary Figure S3B), which inferred two genetic clusters of *H*. *compressa* (Figure 2). Cluster 1 consisted of accessions from Turkana while cluster 2 consisted of accessions from Tharaka, Kwale and Tana River for both reference-based and *de_novo*-based assemblies. The accessions in Cluster 2 were sampled along the Tana basin and Kwale county as shown in Figure 1. The expected heterozygosity was lower for the *de_novo*-based assembly for cluster 1 (*H*_*e*_ = 0.14) than cluster 2 (*H*_*e*_ = 0.23). However, similar expected heterozygosity values were observed in the reference-based assembly for the two clusters (*H*_*e*_ = 0.30). The genetic variation among populations in Cluster 1 was higher (*de_novo F*_*ST*_ = 0.68 and reference-based *F*_*ST*_ = 0.17) than Cluster 2 (*de_novo F*_*ST*_ = 0.3 and Reference-based *F*_*ST*_ = 0.06). A total of seven accessions from Kwale had admixed ancestry between Cluster 1 and Cluster 2 using the reference based assembly (Table 1). There were no accessions in the *de_novo* assembly that had admixed ancestry values less than 88%. Structure results indicate that two gene pools best describe the population structure of *H. compressa*. However, a smaller peak observed at *K* = 3 might be another informative *H. compressa* population clustering (Supplementary Figures S3, S4). Similar grouping of accessions using PCA for the *de_novo* and reference-based assemblies was observed in this study (Figure 3). In both PCA plots, Tharaka Nithi, Kwale and Tana River accessions were closely clustered. PCA results were congruent with structure results.

**TABLE 1:** STRUCTURE analysis of *Hyphaene compressa* from Kenya using reference-based and *de_novo* based assembly.

Assembly method	Tharaka	Tana river	Kwale	Turkana	*He*	*F*_*ST*_
**Reference assembly**	—	—	—
Cluster 1				21	0.29	0.17
Cluster 2	27	20	21	—	0.30	0.06
Admixed	—	—	7	—	—	—
***De_novo* assembly**						
Cluster 1	—	—	—	21	0.14	0.68
Cluster 2	27	20	28	—	0.23	0.30

**FIGURE 4:**
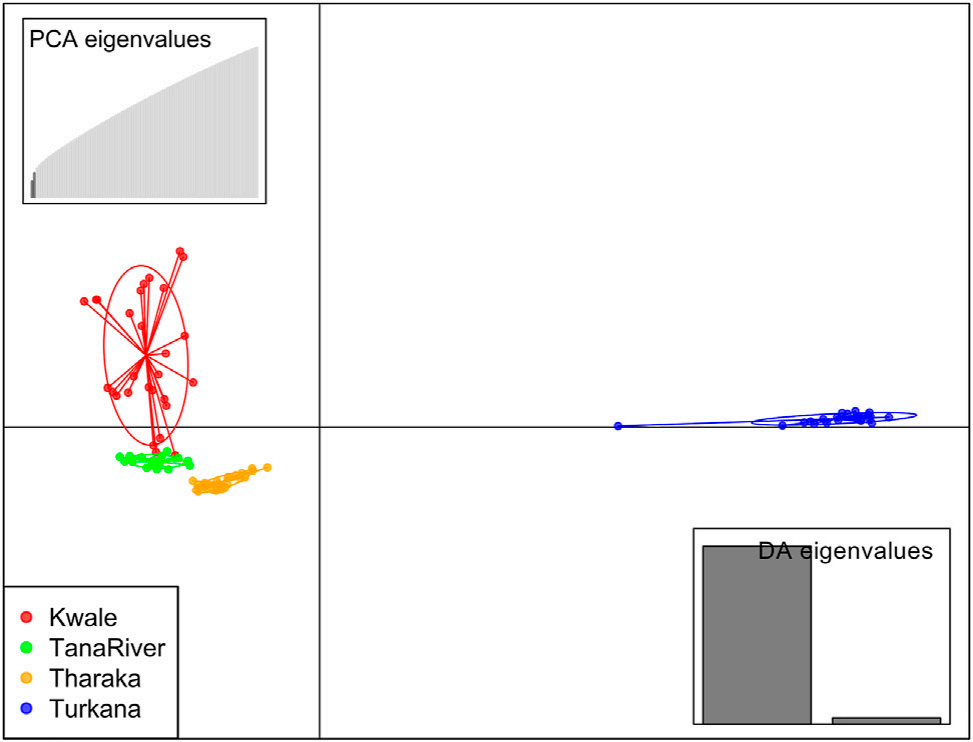
Discriminant analysis of principal components (DAPC) of 96 *Hyphaene compressa* Kenyan accessions using 23,416 SNPs derived from GBS analysis using reference-based assembly. Inertia ellipses represent clusters in different colors. Each dot on the circle represents an accession.

**FIGURE 5:**
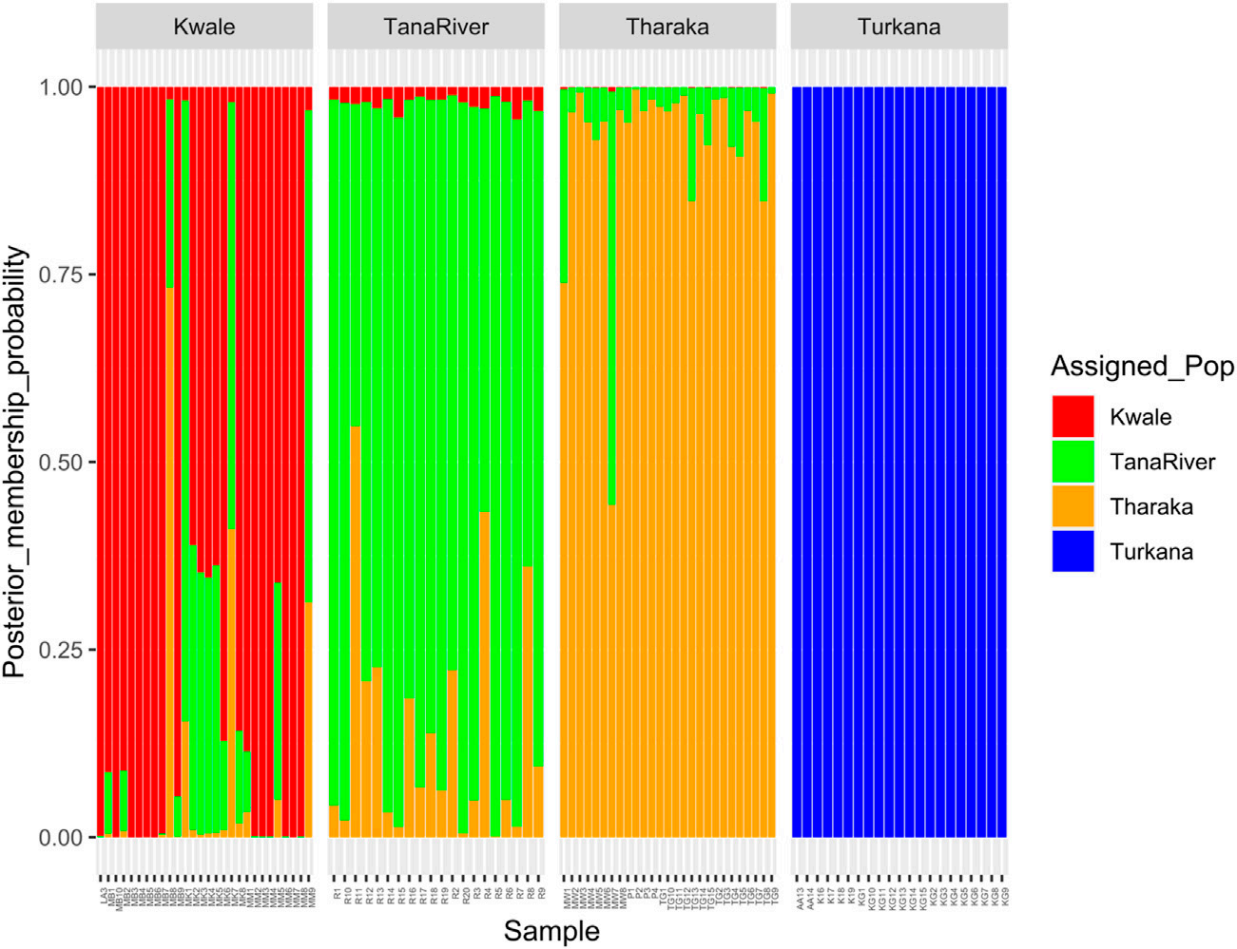
Composite plot of *Hyphaene compressa* Kenyan accessions using reference-based assembly showing mixed ancestry between Kwale, Tana River and Tharaka. Each accession is a stacked bar chart with populations shown in colors.

DAPC analysis grouped *H. compressa* accessions into two clusters, with samples from Turkana falling to the right side of the DAPC vertical axis while the rest fell on the left side. There was moderate overlap between Kwale and Tana River accessions, while Tharaka accessions were distinctly separate (Figure 4). DAPC analysis, composite plot and genetic diversity results are shown for only the reference based assembly SNP data since both assembly methods had shown congruent results in structure and PCA analysis. Population membership assignment using DAPC composite plot confirmed structure and PCA results. All the accessions along the Tana basin exhibited admixture with profoundly shared ancestry between Tharaka and Tana River. Kwale had the highest level of admixture (Figure 5). DAPC results also confirmed no admixture between Turkana and accessions from the other regions.

The four sampled regions of Tharaka, Tana River, Kwale and Turkana were assessed for the number of polymorphic sites, expected heterozygosity (*H*_*e*_) or gene diversity, observed heterozygosity (*H*_*o)*_, *F*_*IS*_ and *F*_*ST*_ *.* The genetic variation among the *H. compressa* populations was moderate (*F*_*ST*_ = 0.074, *p* ≤ 0.001). The observed heterozygosity was higher than the expected heterozygosity in all the populations (Table 2). Negative *F*_*IS*_ values were obtained in all the populations, with Turkana having the lowest *F*_*IS*_ value (−0.45). Kwale had the highest polymorphic sites (11,932) followed by Turkana (10,698) as shown in Table 2. Tana River had the lowest diversity (*H*_*e*_ = 0.23, Polymorphic sites = 8,370) of all the sampled regions (Table 2).

**TABLE 2:** Mean values of genetic diversity indices determined for *Hyphaene compressa* accessions in the sampled populations.

Genetic index	Region				Overall
	Tharaka	Turkana	Tana river	Kwale	
Number of polymorphic sites	9,277	10,698	8,370	11,932	23,416
Observed heterozygosity (*H*_*o*_)	0.45	0.47	0.46	0.44	0.404
Expected heterozygosity (*H*_*e*_)	0.32	0.33	0.23	0.33	0.31
*F*_*IS*_	−0.40	−0.45	−0.42	−0.37	−0.040
*F*_*ST*_					0.074

Pairwise *F*_*ST*_ values ranged between 0.025 (Tharaka and Tana River) and 0.105 (Turkana and Tana River). High pairwise *F*_*ST*_ was recorded among Turkana and Tana River samples (Table 3). The lowest pairwise *F*_*ST*_ was between Tharaka and Tana River, suggesting gene flow between the two regions. AMOVA showed that populations from each of the four regions of Turkana, Tharaka, Tana River and Kwale were slightly different from each other (*p* ≤ 0.001, Table 4). The variation within populations was higher (92.7%) than among populations (7.3%).

**TABLE 3:** Pairwise *F*_*ST*_ values of Kenyan populations of *Hyphaene compressa*.

Population	Turkana	Kwale	Tharaka	Tana river
Turkana				
Kwale	0.07952			
Tharaka	0.09795	0.03629		
Tana River	0.10541	0.03329	0.02505	0.00

**TABLE 4:** Analysis of molecular variance (AMOVA) in genetic variation of *Hyphaene compressa* among and within populations of Kwale, Tharaka, Turkana and Tana River counties in Kenya.

Source of variation	Sum of squares	Variance components	Percentage variation	*p*-value
Among populations	42728.129	266.91865	7.32597	0.00
Within populations	558905.557	3376.54026	92.67403	
Total	**601633.686**	**3643.45891**	**100**	

### Phylogenetic Analysis

**FIGURE 6:**
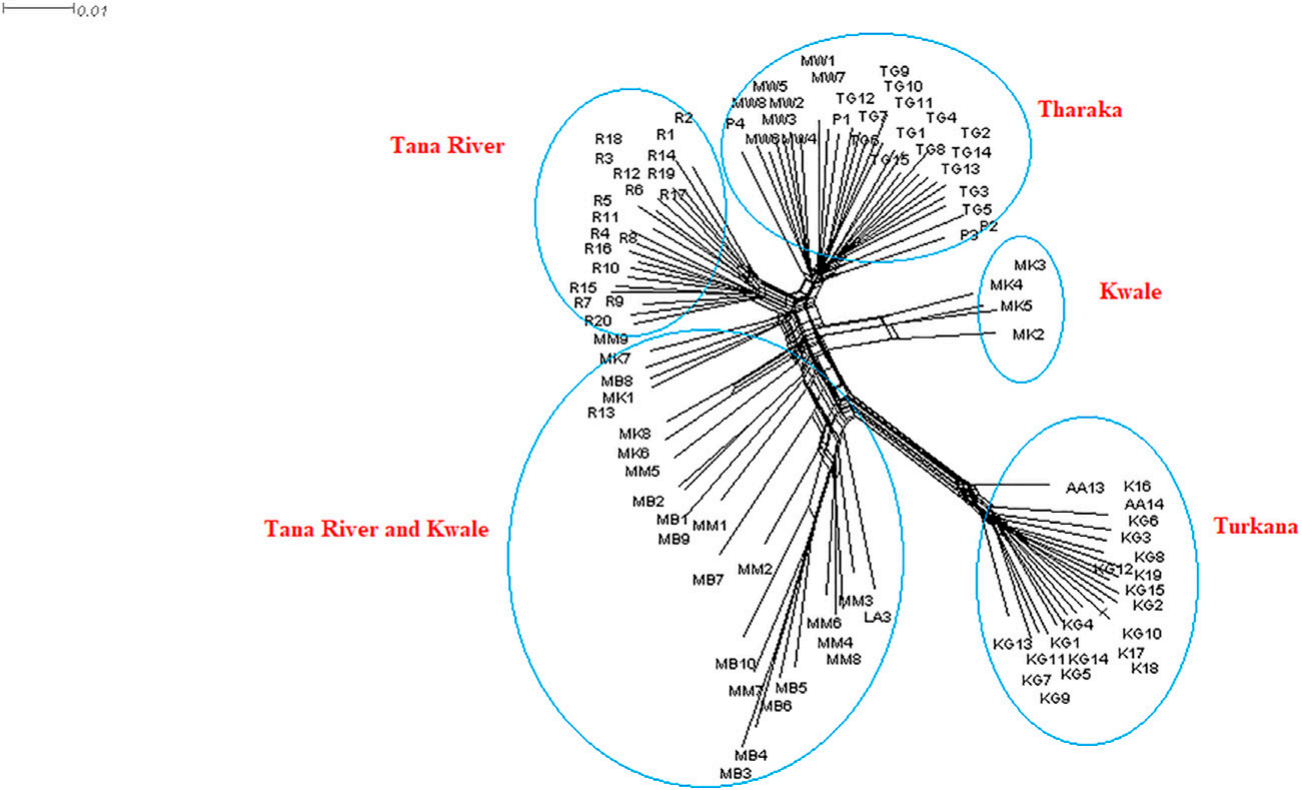
NeighbourNet network generated using 23,416 SNPs derived from reference-based GBS analysis of Kenyan *Hyphaene compressa* using SplitsTree version 4.17.0.

The neighbor net network was able to group *H. compressa* accessions by region. Tana River, Kwale and Tharaka samples clustered closely compared to the Turkana, which was separated from the rest (Figure 6). Samples from Turkana clustered together. Some of the Kwale accessions clustered closely with Tana River while other Kwale accessions clustered more closely with Tharaka accessions. The UPGMA phylogenetic tree showed two main clusters with Turkana accessions clustering in one cluster and the rest of the accessions in the other cluster. Kwale populations also revealed reciprocal monophyly (Supplementary Figure S5).

### Migration Rates Among *H. compressa* Accessions Along the Tana Basin

The highest gene flow was observed from Tharaka and Tana River samples (*m* = 139.1), followed by Kwale to Tharaka (102.7), Tana River to Kwale (63.1), Tana River to Tharaka (59.9), Tharaka to Kwale (50.3) and Kwale to Tana River (57.7). These results indicate that the gene flow along the Tana basin was mostly asymmetrical (Supplementary Figure S6).

## Discussion

This study is the first to report the use of SNPs through the GBS approach to characterize *H. compressa* accessions in Kenya*.* SNP markers are very stable, frequent and specific to regions of the genome which makes them ideal for use in marker assisted selection (MAS) and diversity studies to aid future germplasm conservation.

Two comparative methods (reference-based and *de_novo*-based approaches) were used to infer the population structure and genetic diversity of *H*. *compressa*. In the reference-based assembly, *Phoenix dactylifera* was used as a reference genome. This confamilial genome was used because *H. compressa* had no assembled genome at the time of this study. In the absence of a reference genome of the same species (conspecific) or genus (congeneric), a confamilial reference genome can be used to provide similar estimates of diversity (Brandies et al., 2019; Galla et al., 2019). Galla et al. (2019) further recommends using a confamilial reference genome as the most distant genome ideal for diversity studies. There were differences in the two methods concerning abundance, quality scores and the TS/TV ratios of the SNPs obtained. For example, the highest number of SNPs was observed from the reference-based assembly (23,416) compared to the *de_novo*-based assembly (2096). The reference-based assembly has also been previously demonstrated to outperform *de_novo* assembly in determining the number of SNPs in olive cultivars (D’Agostino et al., 2018). Elsewhere, it has been reported that parameters set during assembly and the type of assembly influence the number and depth of SNPs obtained (Bohling, 2020). Besides, more stringent parameters are normally used for *de_novo* assemblies. GBS of *H. compressa* accessions showed considerable SNP variations with transition SNPs (purine-purine or pyrimidine-pyrimidine) being the most frequent mutations. This high frequency in transition SNPs has also been observed in many plants such as *Capsicum annuum*, *Vigna unguiculata*, *Elaeis guineensis*, and *Camelia sativa* (Pootakham et al., 2015; Taranto et al., 2016; Xiong et al., 2016; Luo et al., 2019; Hyun et al., 2020), of which the C-T transitions are the most frequent (Edwards et al., 2007). However, low TS/TV ratio was observed in *de_novo* based assembly. This phenomenon has also been observed elsewhere and was attributed to the differences in the SNP calling methods (Shafer et al., 2017). Despite the difference between the two methods, structure analysis and PCA produced congruent results. A similar study that used a *de_novo* approach, a confamilial reference and a congeneric reference to determine the phylogenetic relationship of the *Amaranthus* genus, produced differing SNP counts but similar phylogenetic trees (Stetter and Schmid, 2017). Other studies have also reported differing SNP abundance between reference-based and *de_novo* assemblies but consistent population clustering (D’Agostino et al., 2018; Shafer et al., 2017; Stetter and Schmid, 2017).

STRUCTURE analysis grouped the *H. compressa* accessions into two gene pools. PCA and DAPC were consistent with these structure results. Accessions from Turkana (Northern Kenya) were pooled into cluster 1. Cluster 2 had the highest number of genotypes that included accessions collected along the Tana basin sites (Tharaka Nithi and Tana River) and Kwale. In addition, an admixture of accessions was reported for accessions from Kwale that showed mixed ancestry from Tharaka Nithi and Tana River. This admixture may be due to genetic exchange between Kwale, Tharaka Nithi and Tana river. These accessions also exhibited reciprocal monophyly based on phylogenetic analysis. This corroborates the phenotypic diversity studies on *H. compressa* where accessions from Kwale exhibited the highest phenotypic diversity compared to other regions (Omire et al., 2020b).

The fixation index (*F*_*ST*_) is an informative method for measuring population differentiation among populations (Nassiry et al., 2009). An overall *F*_*ST*_ value of 0.074 was reported for *H. compressa* populations in this study. According to Nassiry et al. (2009), an *F*_*ST*_ of 0–0.05 is considered small, 0.05–0.15 is moderate and 0.15 and above is considered very high. Therefore, *H. compressa* accessions had moderate genetic differentiation. However, *F*_*ST*_, values obtained from STRUCTURE population clustering indicate high genetic differentiation within cluster 1 (accessions from the northern part of Kenya) than cluster 2 (accessions along the Tana Basin). On the other hand, cluster two had higher levels of heterozygosity (*He*) compared to cluster 1. This indicates high diversity in accessions along the Tana basin. The high heterozygosity and lower genetic differentiation between *H*. *compressa* accessions along the Tana Basin (cluster 2) might be due to genetic exchange arising from gene flow. Although Tharaka Nithi is found approximately 163 and 391 miles away from Tana River and Kwale respectively, gene flow between these three counties seems high. This could be due to the flow of the River Tana (Figure 1), which traverses both Tharaka and Tana River counties and possibly serves as means of germplasm dispersion. This could explain why Tharaka samples are close to the Tana River accessions on the PCA and the high mixed ancestry as demonstrated by DAPC and STRUCTURE analysis. River Tana is the longest river and the most important drainage basin in Kenya. The river drains from the Kenyan highlands to the Eastern ASAL plateaus and coastal Kenya (Kitheka and Ongwenyi, 2002). Since *H. compressa* grows in riverine areas, seed dispersal through the river is an important factor influencing the population structure of *H. compressa* at the Kenyan Coast. Seed dispersal is essential for biodiversity conservation by driving plant gene flow, population dynamics and functional connectivity between regions (Traveset and Rodríguez-Pérez, 2018). Systematic seed dispersal favours gene flow, increases genetic diversity and lowers the genetic differentiation among populations (Paschoa et al., 2018). Migration rates using MIGRATE-n indicate that there is asymmetrical gene flow along the Tana basin. This supports the hypothesis that seed dispersal along the Tana River drives the population structure of *H. compressa* along the Coast. In addition, high migration rates were observed between Kwale and Tharaka an observation that is confirmed by phylogenetic analysis whereby some Kwale accessions clustered with Tharaka accessions.

There is restricted gene flow into or out of Turkana, which may cause differentiation of its population from the other populations. This was supported by STRUCTURE analysis, PCA, DAPC and neighbor net network, which clustered Turkana distinctly from the rest of the populations. This differentiation may be attributed to the physical distance between Turkana and the other populations. Isolation of Turkana populations inhibits them from mating with the other populations. Turkana is found in the far-flung northern part of Kenya and is considered 100% dryland with scarce rain fed agriculture (Barrow and Mogaka, 2007). In addition, the selection pressures in Turkana differ from those present in the other regions.

The negative *F*_*IS*_ values obtained for *H*. *compressa* populations indicate low levels of inbreeding, high diversity and moderate connectivity between the populations. This may be influenced by the mating system. *Hyphaene compressa* is a dioecious plant (Stauffer et al., 2014), a condition that favors obligate cross pollination which inturn increases intrapopulation genetic diversity (Paschoa et al., 2018; Muyle et al., 2020). Dioecy is one of the adaptations in plants that promote outbreeding (Charlesworth, 2006). High genetic diversity and low inbreeding in *H. compressa* was also supported by AMOVA results which showed higher (92.7%) within population diversity than among population diversity (7.3%).

The understanding of the genetic diversity and population structure within *H. compressa* provides useful information for future selection and appropriate conservation strategies. High priority should be given to the conservation of all populations with high genetic diversity. The conservation of *H. compressa* must consider the two identified clusters to ensure that the high diversity within populations is retained. This can be achieved by maximum collection and *ex situ* conservation of germplasm especially for cluster 2 which had the most diversity.

## Conclusion

This study was able to show the genetic diversity and population structure of *H. compressa* using the GBS approach. *Hyphaene compressa* in Kenyan ASALs is delineated into two gene pools. Cluster 1 comprising accessions in the north of Kenya while cluster 2 comprising accessions found along the River Tana basin. Further, accessions from the Tana basin are more diverse than those found in the northern part of Kenya. In addition, the results indicate that *H. compressa* accessions are interconnected with high gene flow and moderate genetic differentiation, evidenced by high within-population variation than among population variation. The high within population diversity can be harnessed for future breeding and improvement programs for various adaptive traits in *H. compressa.*

## Data Availability

The sequence data generated from this study are archived in the NCBI SRA under BioProject accession number PRJNA756042 (https://www.ncbi.nlm.nih.gov/bioproject/PRJNA756042/)

## Supplementary Material

Supplementary Figure S1 Single Nucleotide Polymorphism (SNP) qualities after filtering the VCF file obtained from *de_novo*-based assembly of GBS data showing the depth per individual. **(A)**, observed heterozygosity **(B)**, Mean depth **(C)** and frequency of missing data per individual **(D)** for *Hyphaene compressa* accessions from Kenya.

Supplementary Figure S2 Single Nucleotide Polymorphism (SNP) qualities after filtering the VCF file obtained from reference-based assembly of GBS data showing the depth per individual. **(A)**, observed heterozygosity **(B)**, Mean depth **(C)** and frequency of missing data per individual **(D)** for *Hyphaene compressa* accessions from Kenya.

Supplementary Figure S3 Optimal Delta k values for different k values inferred during STRUCTURE analysis of Kenyan Hyphaene compressa showing the optimal delta k at k = 2 for the reference-based assembly **(A)** and the de_novo based assembly **(B)**.

Supplementary Figure S4 STRUCTURE bar plot of admixture model population assignment of 96 *Hyphaene compressa* accessions from Kenya showing three clusters based on 2096 Single Nucleotide Polymorphisms (SNPs) for the de-novo assembly **(A)** and 23416 SNPs based on reference assembly **(B)**. A combination of different colors represents admixed populations. In these structure plots, Tharaka and Tana River accessions have been placed in one cluster.

Supplementary Figure S5 Unrooted UPGMA distance tree inferred using 23416 SNPs based on the reference based assembly of *H. compressa* accessions.

Supplementary Figure S6 Migration rates between Tharaka, Tana River and Kwale using MIGRATE-n software. The direction of the arrow indicate the direction of gene flow.
