# Supplementary figures and images for: Genetic Diversity and Population Structure of Doum Palm (Hyphaene compressa) Using Genotyping by Sequencing
Source: Front Genet. 2022 Feb 4;13:762202. doi: 10.3389/fgene.2022.762202 (PMC8854861; doi:10.3389/fgene.2022.762202)

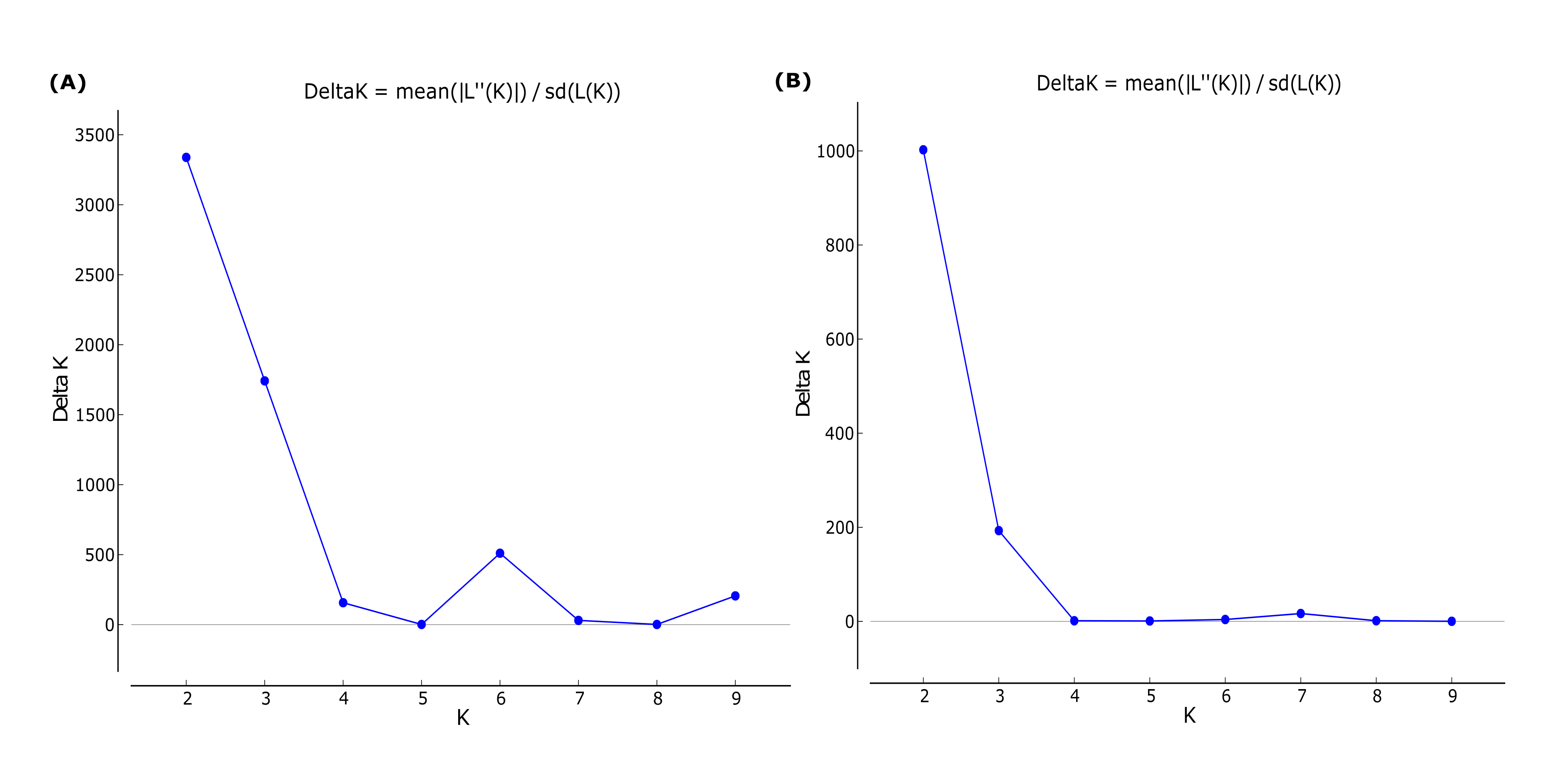

Supplement: Supplementary file 1 [file Image3.TIFF]

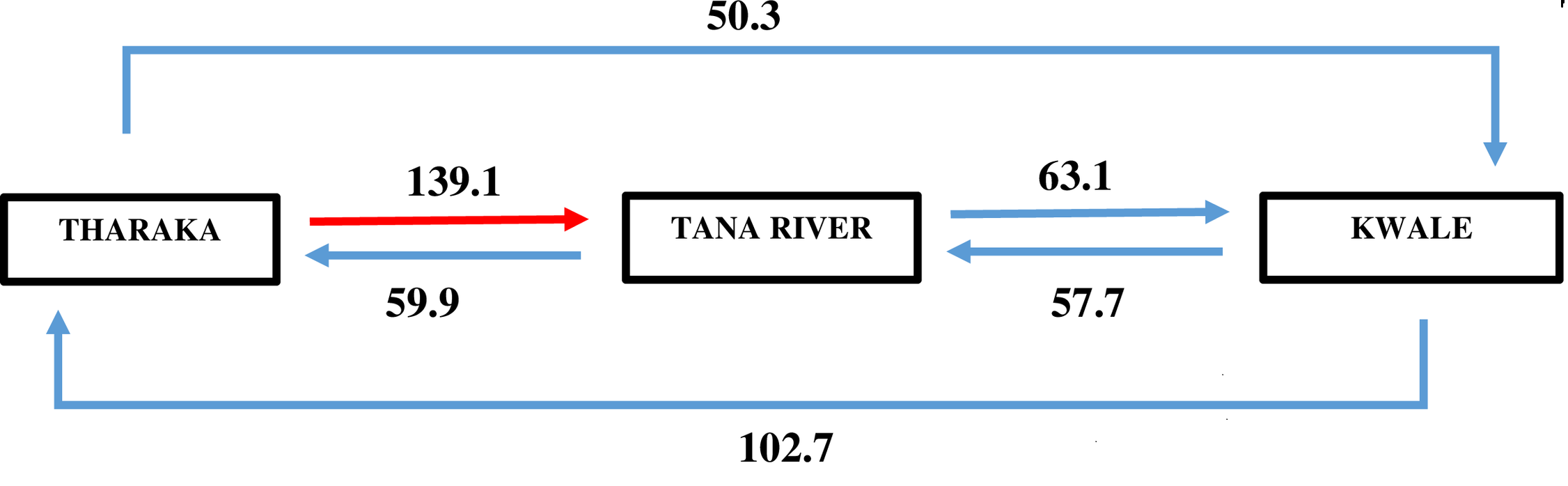

Supplement: Supplementary file 2 [file Image6.tif]

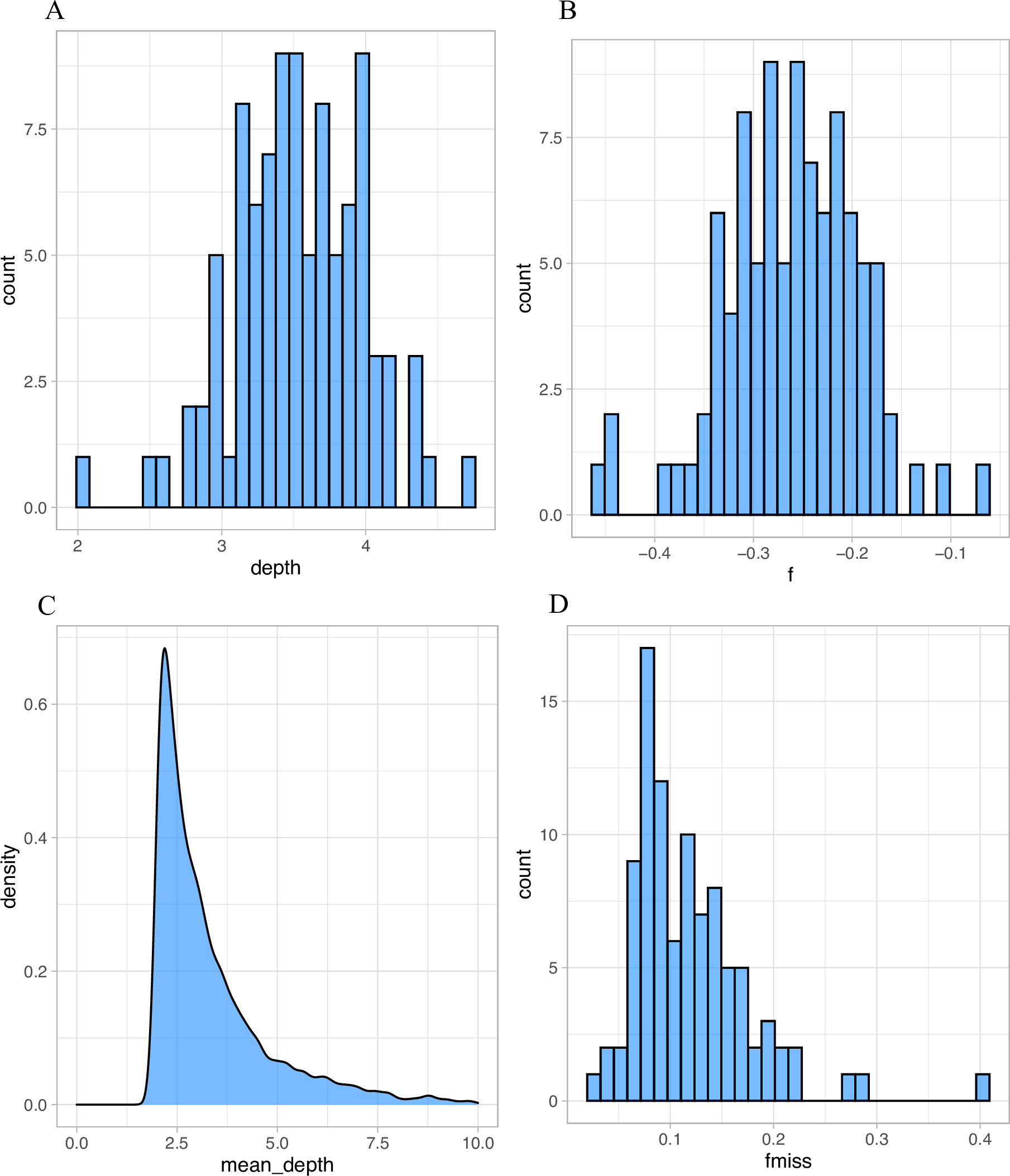

Supplement: Supplementary file 3 [file Image2.TIF]

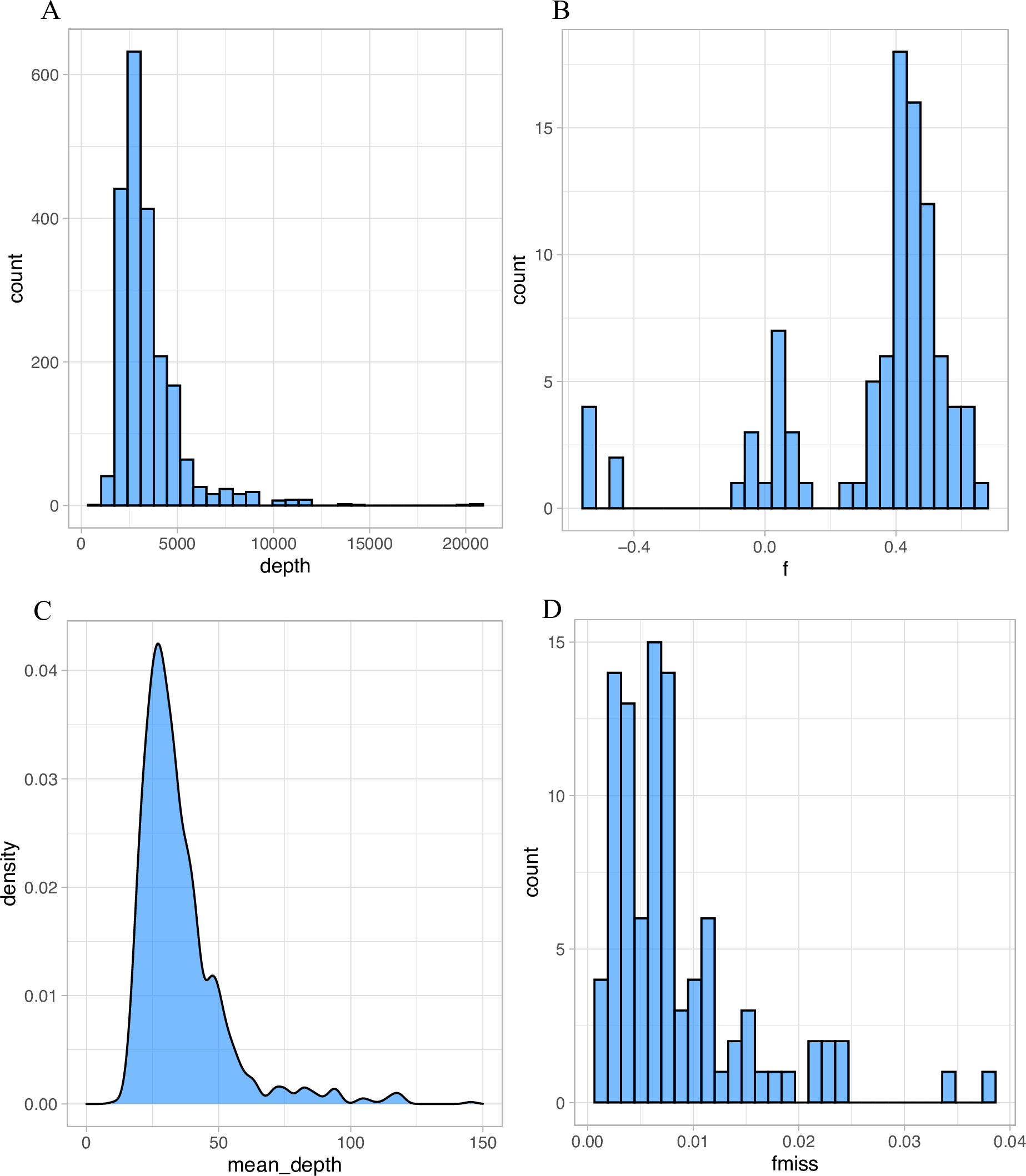

Supplement: Supplementary file 4 [file Image1.TIF]

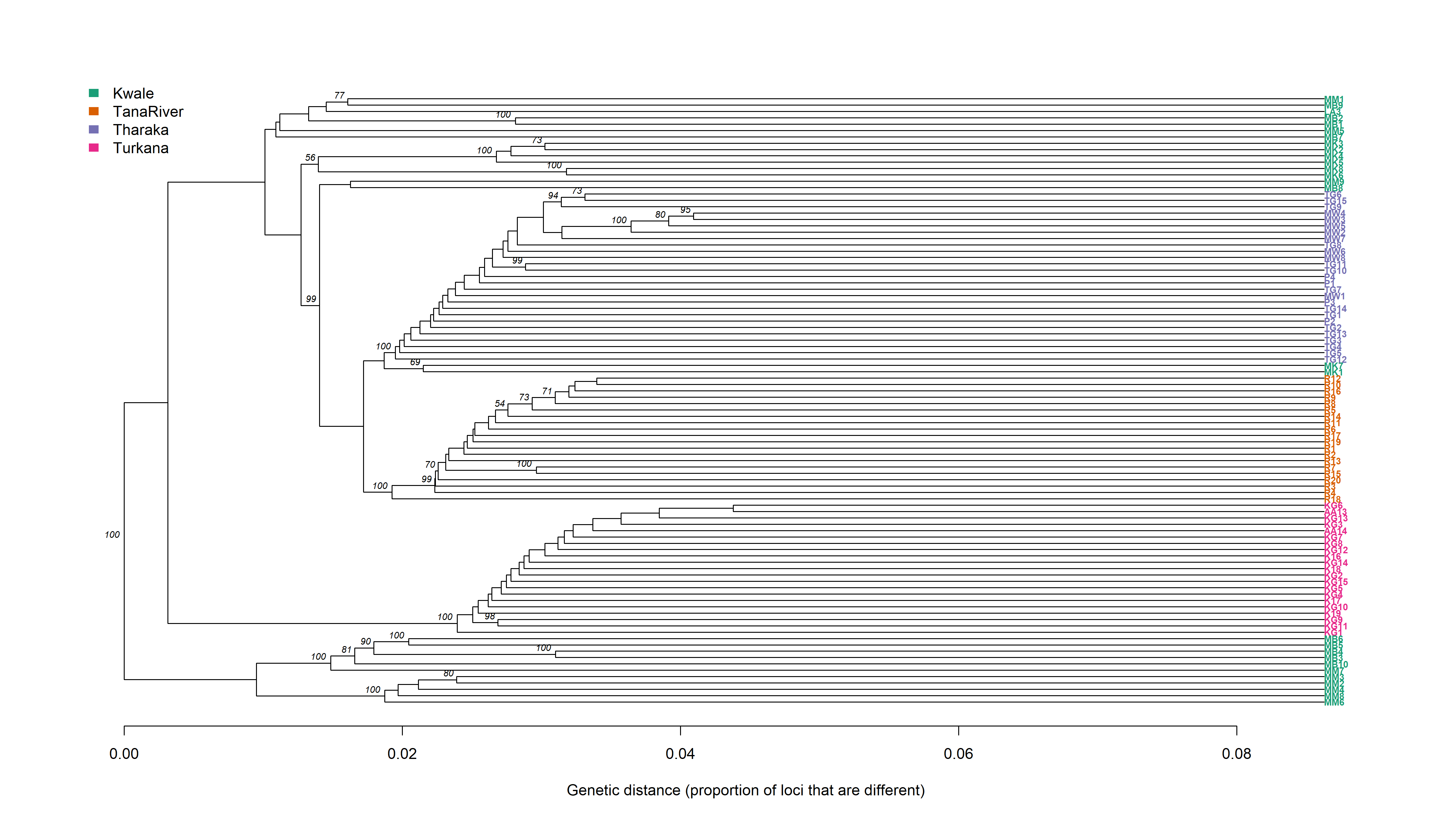

Supplement: Supplementary file 5 [file Image5.png]

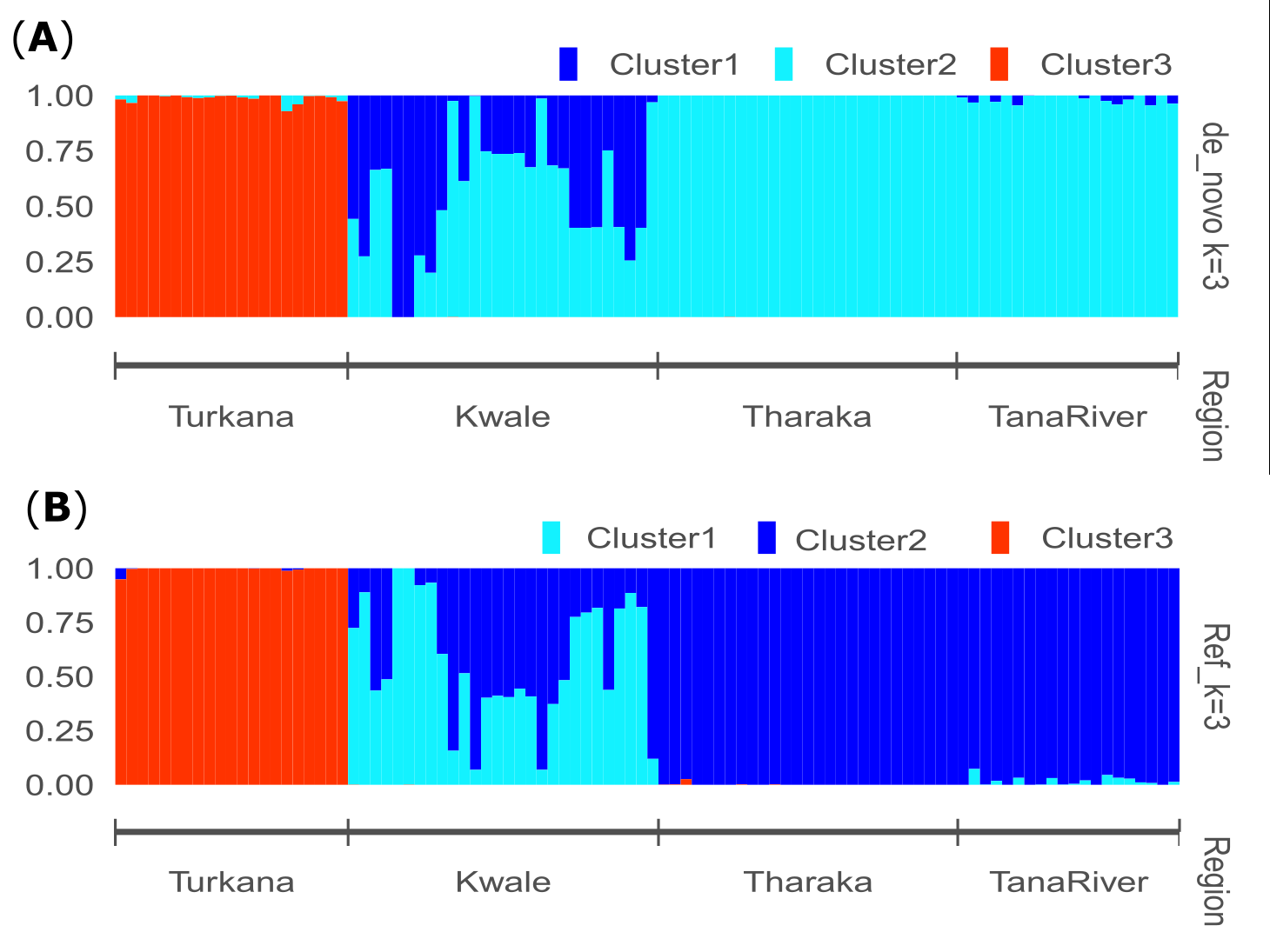

Supplement: Supplementary file 8 [file Image4.TIFF]
