# Supplementary material for: Genetic Diversity and Population Structure of Doum Palm (Hyphaene compressa) Using Genotyping by Sequencing
Source: Front Genet. 2022 Feb 4;13:762202. doi: 10.3389/fgene.2022.762202 (PMC8854861; doi:10.3389/fgene.2022.762202)
Supplement: Supplementary file 6 [file Table2.DOCX]

**Supplementary Table 2**: Transition and transversion events of GBS analysis of *Hyphaene compressa* from Kenya using both the *de_novo* and reference approach.

| **SNP Type** | ***De_novo* assembly**  **Total (Percentage)** | **Reference-based assembly**  **Total (percentage)** |
| --- | --- | --- |
| **Transitions** | 1283 (61.2) | 16598 (70.9) |
| A↔G | 651 (31.1) | 8332 (35.6) |
| C↔T | 632 (30.2) | 8266 (35.3) |
|  |  |  |
| **Transversions** | 813 (38.8) | 6818 (29.1) |
| A↔C | 192 (9.2) | 1684 (7.2) |
| A↔T | 174 (8.3) | 1825 (7.8) |
| C↔G | 222 (10.6) | 1636 (7) |
| G↔T | 225 (10.7) | 1673 (7.1) |
